# Prevalence and Determinants of Drug-Resistant Tuberculosis (DR-TB) Among Tuberculosis Patients in Pokhara Metropolitan City, Gandaki Province, Nepal

**DOI:** 10.1155/ipid/3730719

**Published:** 2025-07-07

**Authors:** Shiv Kumar Sah, Chetan Karki Pyakurel, Arun Kathariya, Anil Shrestha, Nirmal Kumar Subedi, Niru Byanjankar, Rojina Basnet

**Affiliations:** ^1^Department of Pharmacy, Institute of Medicine (IOM), Maharajgunj Medical Campus (MMC), Tribhuvan University, Kathmandu, Nepal; ^2^Department of Pharmacy, Little Buddha College of Health Science, Purbanchal University, Kathmandu, Nepal; ^3^Central Department of Public Health (CDPH), Institute of Medicine (IOM), Tribhuvan University, Kathmandu, Nepal

**Keywords:** antitubercular drugs, associated factors, drug resistance-tuberculosis, prevalence

## Abstract

**Background:** Drug-resistant tuberculosis (DR-TB) remains a significant global public health challenge, particularly in regions with a high burden of TB. Nepal, one such country, has been witnessing a rise in DR-TB cases, posing serious challenges to TB control efforts. Despite this growing concern, there is a lack of localized data on the risk factors contributing to DR-TB, especially in urban areas like Pokhara. This study aims to fill that gap by assessing the prevalence of DR-TB and identifying associated demographic, behavioral, and clinical factors among TB patients in Pokhara Metropolitan City, Gandaki Province, Nepal.

**Methods:** A retrospective cross-sectional analysis was conducted using 617 TB patient records from the Pokhara Metropolitan Health Office for the fiscal year 2078/79 (July 2021 to July 2022). Data on demographic characteristics, clinical history, treatment regimens, and behavioral factors such as smoking and alcohol consumption were extracted. Descriptive statistics were used to determine the prevalence of DR-TB, and bivariate logistic regression was applied to identify statistically significant risk factors associated with DR-TB.

**Results:** Among the 617 TB patients, the prevalence of DR-TB was 2.6%. Most patients were male (57.4%) and within the 21–30 age group (26.9%). Pulmonary bacteriologically confirmed TB was the most common type (53.6%), predominantly affecting adults (98.1%). The primary treatment regimen administered was 2HRZE + 4HR (78.8%). TB-HIV co-infection was found in 1.9% of cases, with all co-infected patients receiving antiretroviral therapy. In a bivariate analysis, individuals with current smoking status (UOR: 9.384; CI: 3.342–26.351), exposure to smoking (UOR: 8.550: CI: 2.916–25.064), and current alcohol consumption (UOR: 4.553, CI: 1.406–14.745) had a higher likelihood of DR-TB. In a multivariate analysis, exposure to smoking (AOR: 5.317; CI: 1.394–20.274) and current alcohol consumption (AOR: 6.84; CI: 2.071–22.58) emerged as independent predictors associated with an increased risk of DR-TB.

**Conclusion:** The study revealed a relatively low prevalence of DR-TB among TB patients in Pokhara, with strong associations between DR-TB and lifestyle factors such as smoking and alcohol use. These findings underscore the need for targeted public health interventions addressing behavioral risk factors to reduce DR-TB incidence. Enhanced surveillance, public awareness, and preventive strategies should be integrated into TB control programs to mitigate the spread of DR-TB in this region. Moreover, targeted behavioral interventions may be crucial in curbing the emergence of DR-TB, particularly in high-burden urban centers.

## 1. Introduction

Tuberculosis (TB) remains a global health burden [1], contracting 10 million people annually, and despite treatment availability, 1.5 million die from it, with low-and middle-income country being most affected [2].

In Nepal, TB is among the top 10 causes of mortality, with an estimated 45,000 new active TB cases reported annually, of which over 20,000 are infectious [3]. Although the Directly Observed Treatment Short Course (DOTS) strategy has improved outcomes, TB still causes 5000–7000 deaths annually [4].

Drug-resistant TB (DR-TB) poses a global threat, especially in resource-limited countries like Nepal, where marginalization, stigma, high costs, and limited social protection exacerbate the issue [3, 5, 6]. Prevalence of DR-TB varies considerably worldwide, ranging from 2.5% to 33.3% [5, 7–9]. In Nepal, DR-TB ranks among the top 10 causes of death, and claims approximately 17,000 lives each year, and has a significant negative impact on social and economic burden [10]. According to fiscal year annual report 2020/21, in Nepal, 2200 suffer DR-TB, yet only 517 were detected, highlighting a public health threat [4]. Nepal's diagnostic capabilities are inadequate, making it difficult to detect DR-TB, and owing to lack of resources, conducting drug susceptibility tests (DST) for all cases is unfeasible [11].

The risk factors associated with DR-TB are multifactorial, and considerably varies across the study settings. Epidemiological studies suggest that patients previously treated with anti-TB [12–14], poor-quality DOTS [13], long-term treatment [13], age over 40 years [13], diabetes mellitus [12], and intravenous drug use [12] contribute to the development DR-TB. The risk factor responsible for the development of DR-TB could be different in Nepal than those cited in the literature due to geographical, cultural, and socioeconomical variation in the country. However, the data on the risk factors of DR-TB in Nepal is limited, and Nepal's National TB Programme faces challenges due to DR-TB, warranting investigation into associated factors [11]. To develop effective interventions and programs, understanding the existing burden of DR-TB, medication practice, and factors linked to DR-TB is crucial. Insights from this study can contribute to public health policies aimed at reducing DR-TB incidence and improving TB treatment outcomes. Despite the recognized burden of DR-TB in Nepal, data on its associated risk factors remain scarce. Given the country's diverse sociocultural and geographic landscape, these factors may differ from global trends. Thus, this study aims to estimate the prevalence of DR-TB and identify demographic, behavioral, and clinical factors associated with its occurrence among TB patients in Pokhara Metropolitan City.

## 2. Methods

### 2.1. Research Design and Setting

This retrospective cross-sectional study reviewed outpatient department (OPD) records of TB patients from Pokhara Metropolitan City for the fiscal year 2078/79 (corresponding to mid-July 2021 to mid-July 2022 in the Gregorian calendar). OPD records of all TB patients during this period were examined. To ensure data confidentiality, each record was assigned a unique code for de-identification. A standardized checklist, developed based on the study objectives, was used to maintain data uniformity. Only records with complete information were included in the analysis.

#### 2.1.1. Study Site

The study was conducted in Pokhara Metropolitan City, located in Gandaki Province, Nepal. Pokhara is a major urban center with a diverse population and a notable prevalence of TB cases, making it an important area for studying TB and DR-TB. Patient records were obtained from the TB register at the Pokhara Metropolitan Health Office, which coordinates all TB-related activities at the municipal level. This office is responsible for providing clinical services for TB, including diagnosis, treatment, and management of DR cases, ensuring comprehensive and reliable data on the prevalence and management of TB in this region. Given the burden of TB in the area, Pokhara offers a representative and accessible setting for examining factors associated with DR-TB and identifying local public health needs.

#### 2.1.2. Study Population and Criteria for Sample Selection

Individuals with all age groups, with a confirmed diagnosis TB whose record was available at Metropolitan Health Office for 1 year were eligible for the study. Those records with insufficient demographic and incomplete information were excluded from the study.

#### 2.1.3. Variables of Interest

The core outcome variable include DR-TB, whereas age, gender, BMI, smoking history, alcohol consumption history, types of TB, TB treatment history, under TB dots program, patient type, treatment regimen, HIV-infection, ART were considered covariates.

#### 2.1.4. Sample Size

This cross-sectional retrospective study included the entire population of interest for the fiscal year 2078/79, so no sample size calculation was necessary. Initially, 700 TB patient records were available in the TB register. However, after applying inclusion and exclusion criteria during data collection, 617 cases met the criteria and were included, making 617 the final sample size for the study.

A total enumeration of all available TB patient records meeting inclusion criteria was conducted, rather than sampling.

### 2.2. Procedures

Approval from the Institutional Review Committee (IRC) of Purbanchal University (Ref: 028A-079/80) was taken. Formal permission was taken from the Health Office of Pokhara Metropolitan City (Ref: 683). After that, the necessary data sets were collected as per the objective of the study. Structured checklist was used to collect data. This study was done on the retrospective data that was already captured in the TB register of the Metropolitan Health Office of Pokhara Metropolitan City based on the checklist.

### 2.3. Laboratory Investigation

All the laboratory investigation was done complying the national TB management guideline, Nepal, and WHO guideline [15, 16]. DR-TB was assessed as per the records available for the sensitivity test done for antitubercular drugs. Among the available records, sensitivity test was conducted only for rifampicin, and based on this information, DR-TB was evaluated accordingly.

### 2.4. Validity and Reliability

To ensure the validity, several measures were implemented. First, we used a complete set of patient records for the fiscal year 2078/79, covering all TB cases recorded in the official TB register of the Pokhara Metropolitan Health Office. This helped provide an accurate representation of the target population and minimized selection bias, enhancing the study's external validity. Additionally, a standardized data collection checklist through extensive literature review was developed based on the study objectives to ensure consistent data extraction. The checklist ensured that only relevant information was captured and that all records met the inclusion and exclusion criteria, supporting construct validity. Moreover, adequate sample size was taken to ensure external validity.

For reliability, each patient record was assigned a unique code to de-identify data, ensuring consistent tracking and analysis of each case. A comprehensive data review process was conducted to confirm that only complete records were included in the analysis, reducing the chance of missing or inconsistent data. The use of routinely collected data from a centralized and regulated TB register also contributed to the reliability of findings, as the data was collected and recorded by trained health professionals following standard procedures.

### 2.5. Data Management and Analysis

Data were analyzed using SPSS version 26.0. Descriptive statistics (mean, SD, frequencies, and percentages) summarized demographic and clinical variables. Chi-square or Fisher's exact tests were used for categorical comparisons, and *t*-tests or Mann–Whitney *U* tests for continuous variables. Variables with *p* < 0.05 in bivariate analysis were included in multivariate logistic regression to identify independent predictors of DR-TB. Odds ratios (ORs) with 95% confidence intervals (CIs) were reported. Model fit was assessed using the Hosmer–Lemeshow test, and discriminatory ability evaluated through ROC curve analysis, with area under the curve (AUC) > 0.7 considered acceptable.

### 2.6. Ethical Consideration

The study adhered to the ethical principles of the Declaration of Helsinki. Approval was obtained from the IRC of Purbanchal University (Ref: 028A-079/80), and formal permission from the Pokhara Metropolitan City Health Office (Ref: 683). All data were anonymized by assigning unique codes to maintain confidentiality. Only de-identified and complete records were used for analysis.

## 3. Results

Table 1 presents the sociodemographic characteristics of the study participants. Results showed a male majority (57.4%) among participants, while females comprised 42.6%. The largest age group was 21–30 years (26.9%), with a mean age of 38.33 ± 20.07 years. Over half of the participants (56.7%) had a healthy BMI (18.5–< 25), 21.4% were classified as overweight, 18.3% were underweight, and 3.6% were obese.

Table 2 summarizes the behavioral characteristics of the study participants. Results indicate that 8.6% of TB patients were current smokers, 16.4% had some exposure to smoking, and 5.7% reported alcohol use.

Table 3 demonstrates the TB-related clinical characteristics of the patients. Regarding TB type, more than half (53.6%) of patients had pulmonary bacteriologically confirmed (PBC) TB, 30.6% had extra pulmonary (EP) TB, and 15.7% had pulmonary clinically diagnosed (PCD) TB. Only 4.7% were in the community-based DOTS program. Most cases (98.1%) were adults, and the predominant treatment regimen was 2HRZE + 4HR (78.8%). TB-HIV co-infection was seen in 1.9% of patients, all receiving antiretroviral therapy (ART).

Table 4 illustrates the prevalence of DR-TB in the studied population. Among the analyzed sample, the estimated prevalence of DR-TB was 2.6% (95% CI: 1.34%–3.86%).

The results on the association between DR-TB and sociodemographic and TB-related clinical factors are shown in Table 5. Test for significance using Chi-square revealed a significant association of DR-TB with current smoking status, exposure to smoking and current alcohol (*p* < 0.05).

Table 6 shows the adjusted and unadjusted OR for the determination of the independent factors associated with the development of DR-TB. In a bivariate analyses, TB-HIV co-infection (OR: 8.443, CI 1.691–42.155) showed a significant relationship with DR-TB. Behavioral factors such as current smoking status, exposure to smoking, and current alcohol demonstrated significant risk factors for the DR-TB. Specifically, individuals with current smoking status had a significantly higher likelihood of DR-TB (OR: 9.384, CI 3.342–26.351), exposure to smoking was linked to a higher risk of DR-TB (OR: 8.550, CI 2.916–25.064), and current alcohol consumption was significantly associated with DR-TB (OR: 4.553, CI 1.406–14.745), HIV infection (OR: 8.443, CI: 1.691–42.155). In a multivariate analysis, exposure to smoking (AOR: 5.317, CI: 1.394–20.274) and current alcohol consumption (AOR: 8.84, CI: 2.07–22.58) still emerged as independent predictors of DR-TB.

**Figure 1:**
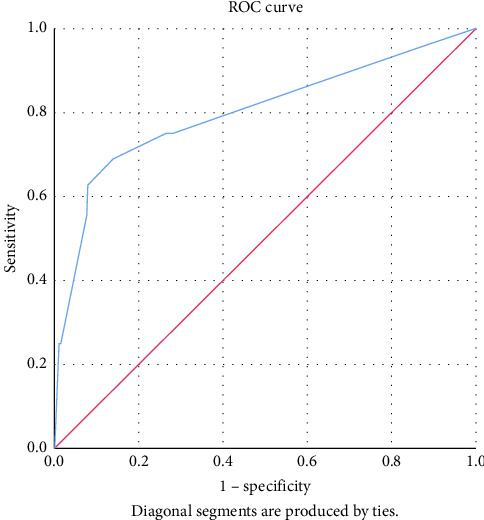
Receiving operating characteristic (ROC) curve for robustness of the model.

The Hosmer–Lemeshow goodness-of-fit test showed a predicted value of 97.2% (*p*=0.17) indicating the robustness of our logistic regression model, and the area under the ROC curve was 0.801; 95% CI: 0.66–0.93, suggesting that the model had good discriminatory ability (Figure 1).

## 4. Discussion

The present study utilized a cross-sectional retrospective record review design to examine the prevalence of DR-TB and its associated factors among TB patients in Pokhara Metropolitan City during the fiscal year 2078/79. The findings highlight the ongoing burden of DR-TB and provide numerous key insights that are crucial for the development of targeted interventions and public health programs aimed at combating this growing threat. This study revealed a DR-TB prevalence of 2.6% in Pokhara Metropolitan, a rate lower than previous national and global estimates. Behavioral factors such as smoking and alcohol consumption emerged as significant predictors of DR-TB. Individuals exposed to smoking and current alcohol consumers showed notably higher odds of resistance, the overall prevalence of DR-TB in this study was found to be 2.6%. This prevalence aligns closely with findings from other studies conducted in various regions, including 3.2% in central Nepal [17], 2.5% [7] and 3.4% in Ethiopia, and 4.2% in North-Western Nigeria [8].

However, this figure is notably lower than the 10.2% [5] prevalence reported in an earlier study in Nepal, as well as the broader global range of 5.7%–33.3% observed in different studies [7, 9, 18–20]. The lower prevalence of DR-TB observed in this study may be attributed to the fact that drug resistance was assessed solely based on rifampicin susceptibility testing, whereas other studies often considered resistance to rifampicin, isoniazid, or other first-line antitubercular drugs limited use of molecular tests beyond rifampicin resistance) might have led to underestimation of true DR-TB prevalence. Additionally, discrepancies in prevalence rates could stem from differences in patient selection, sample size (as smaller samples may yield higher resistance rates), treatment protocols, control measures, and patient adherence to treatment. Variations in the level of awareness about drug resistance within the population and disparities in access to healthcare facilities may also contribute to these differences in reported prevalence.

The study identified a higher prevalence of male participants (57.4%) compared to females (42.6%). Consistent trends observed have been observed in studies conducted in Pakistan [21], China [22], and Mali [23], also [16, 18, 19]. This gender distribution may demand further investigation to understand underlying factors contributing to this difference in TB incidence between genders.

Age distribution among TB patients revealed that majority (26.9%) were within the age range of 21–30 years. This demographic concentration within the young adult age group is consistent with the findings from similar studies in Asian countries, including India [2], China [22], and Thailand [24]. This shared age-specific pattern might be attributed to a complex interplay of factors, such as lifestyle choices, living conditions, and social interactions, which are likely prevalent across these Asian societies. Further investigation into these factors could identify targeted interventions tailored to this particular age cohort.

With regards to the nutritional status, the prevalence of overweight and underweight in the study patients were notable, with 21.4% and 18.3%, respectively. Additionally, a smaller percentage (3.6%) of patients was classified as obese. Available evidence reveals a considerable variability in nutritional status among TB patients. For instance, a recent meta-analysis by Li et al. [25], reported a malnutrition prevalence of 48%, while a systematic review and meta-analysis in Ethiopia found a higher proportion of underweight TB patients (50.8%) compared to the current study [26]. The percentage of patients with a healthy weight (BMI 18.5 to < 25) was lower (47.3%). These findings stresses the importance of considering nutritional factors in TB management and the presence of both underweight and overweight patients highlights the importance of addressing nutritional needs as part of comprehensive TB care.

Regarding TB type, PBC TB was the most prevalent form, accounting for 53.6% of cases, followed by EP TB at 30.6% and PCD TB at 15.7%. In earlier study [27], a similar distribution of TB types were reported, with 51.3% PBC TB, 31.9% EP TB, and 16.8% PCD TB [15]. These results show consistency in the proportion of TB types between the two studies, highlighting the predominance of bacteriologically confirmed TB cases in both populations. However, in another study conducted in Mali [23] showed a higher proportion of PBC TB cases (62.1%) and a lower percentage of EP TB cases (18.9%) compared to the current study. which also conforms the findings from India [28]. In a cross-sectional survey conducted by Kang et al. [22] in China, the distribution of TB types was different, with a higher percentage of EP TB cases (46.2%) and lower proportions of PBC TB (39.3%) and PCD TB (14.5%). This suggests that the clinical presentation of TB may vary significantly between countries, possibly influenced by factors such as the healthcare system, diagnostic practices, and population characteristics.

The study revealed that the majority of TB cases (98.1%) belonged to the adult age group, signifying the higher susceptibility of adults to TB infection. This is also supported by the earlier studies conducted inside and outside the country. For instance, a study from Yemen [28] revealed that adults aged 15–45 years were the most affected group by TB, constituting 58.5% of the cases. Although this study reported a slightly lower proportion of adult TB cases compared to the current research, the trend of higher susceptibility in the adult age group remains evident. Sinshaw et al. in Ethipoia [29] reported that the age group of 26–45 years was significantly associated with multi-DR-TB (MDR-TB). Similar results were observed in Mali and another study of Nepal [19, 23]. This finding is in line with the current study, which highlights the higher vulnerability of adults to TB infection and its potential progression to DR forms. Moreover, this finding highlights the ongoing burden of TB/HIV co-infection and support the need to strengthen integrated service delivery. The study also underscore the importance of routine TB screening for all HIV-positive clients and vice versa, strengthening adherence counseling and support mechanisms, enhancing provider training on TB/HIV co-management.

Treatment regimens among TB patients in this study were varied, with the 2HRZE + 4HR regimen being the most frequently prescribed (78.8%), followed by the 6HRZE regimen and the 2HRZE + 7HRE regimen. A smaller percentage of patients received the 6HRZE + Lfx regimen. A study by Mehari et al. [30] in Tigray Region, Ethiopia, reported an even higher prevalence of the 2HRZE + 4HR regimen (89.1%) than seen in this study. Their study also found the 6HRZE regimen to be relatively common (6.4%). These differences in treatment practices may reflect variations in regional TB management guidelines and drug availability. Similarly, research conducted in Yemen by Jaber and Ibrahim [28] also identified the 2HRZE + 4HR regimen as the most commonly prescribed (88.4%), with less frequent use of the 6HRZE + Lfx regimen, consistent with the present study's findings. The preference for the 2HRZE + 4HR regimen across different settings suggests that it is widely accepted and implemented internationally. In contrast, a study from Punjab, Pakistan, by Ullah Irfan et al. reported the 2HRZE + 4HR regimen as the most commonly used (62.2%), followed by the 2HRZE + 2HR regimen (18.3%), with the 6HRZE and 2HRZE + 7HRE regimens being less frequently prescribed. These variations in treatment regimens across countries may stem from differences in drug resistance patterns, local TB management guidelines, and patient-specific factors, such as underlying health conditions and tolerance to medications. The observed consistency in favoring the 2HRZE + 4HR regimen underscores its general effectiveness and adaptability to different clinical contexts.

TB/HIV co-epidemic remains a major public health burden in many parts of the world, and the prevalence of HIV/TB co-infection is diversified among the countries. A systematic review and meta-analysis performed by Gao et al. [31] noted that TB/HIV co-infection prevalence ranged from 2.93% to 72.34%, with the random effects pooled prevalence of TB/HIV co-infection was 23.51%. In this study, TB-HIV co-infection was observed in 1.9% of the TB patients, and all co-infected individuals were ART for their HIV infection. Therefore, the present study underscores the need of HIV surveillance program among TB patients and highlights the importance of integrated TB-HIV management strategies to improve outcomes for co-infected patients.

Our study identified a statistically significant association between HIV-TB co-infection and DR-TB. Through univariate analysis, we observed that individuals with TB-HIV co-infection had a significantly higher likelihood of developing DR-TB compared to those without HIV co-infection (OR: 8.443). This finding may be attributable to the increased frequency and duration of healthcare facility visits among TB/HIV co-infected individuals, which potentially heightens their exposure to circulating DR-TB strains within healthcare settings. The implications of these findings underscore the need for targeted infection control measures and tailored treatment protocols to mitigate the risk of DR-TB in patients with TB-HIV co-infection.

Our study demonstrated significant statistical associations between behavioral factors—such as smoking status, and current alcohol consumption—and DR-TB. Specifically, individuals exposed to smoking and those who currently consume alcohol showed a significant association with DR-TB. Epidemiological studies have similarly identified a higher prevalence of smoking among TB patients, noting it as a risk factor for developing DR-TB, with a reported prevalence ranging from 9.8% to 18.2% [27, 32, 33]. In our study, approximately 7% of patients had a history of smoking, which emerged as an independent risk factor for DR-TB (OR: 8.550; 95% CI: 2.916–25.064; *p* < 0.001), aligning with previous findings that smokers are more likely to develop DR-TB [33]. These findings highlight the necessity of targeted smoking cessation and alcohol reduction programs to alleviate the burden of TB and enhance treatment outcomes. Incorporating these lifestyle factors into TB management strategies is essential, as they may influence treatment compliance and the disease's progression.

Alcohol consumption has long been linked to weakened immune function, poorer health outcomes, and lower adherence to medical treatments—all factors generally expected to increase susceptibility to TB [34] and possibly DR-TB. Despite these theoretical connections, the relationship between alcohol use and DR-TB remains an area of uncertainty, with prior research yielding inconsistent results. For example, a retrospective cohort study by Song et al. found no significant impact of alcohol consumption on the development of DR-TB [34]. Contrarily, our study revealed that current alcohol consumers had significantly increased odds of DR-TB. This discrepancy may reflect differences in methodological approaches, such as sample size, geographical variation, or adjustments for potential confounders like socioeconomic status and comorbidities. The significant association observed in our study could be attributed to the compounding effects of alcohol on TB treatment adherence and immune suppression. Alcohol-related behavioral factors, such as missed doses or delays in seeking care, might create conditions conducive to the development of DR strains.

These findings highlight the complex interplay between alcohol use and DR-TB, underscoring the need for further research to elucidate the mechanisms driving this relationship. Future studies should aim to account for potential confounders and investigate the role of alcohol in both the biological and behavioral pathways influencing DR-TB. Public health interventions targeting alcohol use among TB patients may represent a critical component of strategies to reduce the burden of DR-TB, not only in Nepal but also in a global scale.

### 4.1. Limitations of the Study

Despite the valuable contributions of this study, several knowledge gaps remain. For instance, the study did not explore the association between drug resistance and other risk factors, such as previous TB history, contact with known TB patients, and treatment adherence, and co-morbidity. Future research should consider investigating these factors to gain a comprehensive understanding of drug resistance patterns. Moreover, the study only focused on patient-level factors and did not explore the influence of healthcare system factors, socioeconomic status, and health policies on DR-TB. This current study includes the patients from a Gandaki Province, so generalization of whole country will be difficult. Since, this is a cross-sectional study, the time intervals for which the prevalence of DR-TB is assessed may not be a true reflection of what happens all the time. The casual association between the prevalence and predictive factors over the time cannot be determined. A prospective study should be adopted in future studies to capture the complex interplay of individual and contextual factors contributing to the development of drug resistance. Furthermore, records with insufficient demographic information and incomplete data were excluded from this analysis, which may have impacted the accuracy of the reported DR-TB prevalence. Patients with missing data may possess unique risk factors, health behaviors, or socioeconomic backgrounds that could be potentially linked to DR-TB outcomes. As per available records, DST was performed only for rifampicin. Therefore, DR-TB in this study was operationally defined based on rifampicin resistance only, which may underestimate the true burden of MDR-TB.

## 5. Conclusion

This study revealed a relatively low reported prevalence of DR-TB in Gandaki Province. However, this should be interpreted cautiously, as it may reflect underdiagnosis or limited drug resistance surveillance rather than true disease burden. Behavioral factors—particularly smoking and alcohol consumption—were independently associated with DR-TB, indicating that lifestyle behaviors may play a role in increasing the risk of drug resistance. These findings underscore the need to integrate behavioral risk assessment into TB prevention and management strategies. Strengthening DR-TB surveillance systems while implementing targeted interventions, such as smoking cessation and alcohol use reduction programs, could enhance the effectiveness of TB control efforts in Gandaki Province.

## Figures and Tables

**Table 1:** Sociodemographic characteristics of the study participants.

Variables	Inference
*n*	617
Gender (*n*, %)	
Male	354 (57.4)
Female	263 (42.6)
Age, mean ± SD	38.33 ± 20.07
Age group (*n*, %)	
0–10	12 (1.9)
11–20	117 (19.0)
21–30	166 (26.9)
31–40	75 (12.2)
41–50	71 (11.5)
51–60	66 (10.7)
60–70	65 (10.5)
71–80	29 (4.7)
81 and above	16 (2.6)
BMI, *n* (%)	
Underweight	113 (18.3)
Normal	350 (56.7)
Overweight	132 (21.4)
Obese	22 (3.7)

*Note:* BMI classification: underweight < 18.5; normal 18.5–24.9; overweight 25–29.9; obese ≥ 30.

**Table 2:** Behavioral characteristics of TB patients.

Variables	Inference
Current smoking status (*n*, %)	
Yes	53 (8.6)
No	564 (91.4)
Exposure to smoking (*n*, %)	
Yes	101 (16.4)
No	516 (83.6)
Current alcohol consumer (*n*, %)	
Yes	35 (5.7)
No	582 (94.3)

**Table 3:** TB-related characteristics of study participants.

Variables (*n* = 617)	Inference
TB type (*n*, %)	
PBC	331 (53.6)
EP	189 (30.6)
PCD	97 (15.7)
Under CB DOTS program (*n*, %)	
Yes	29 (4.7)
No	588 (95.3)
Patient type (*n*, %)	
Child	11 (1.9)
Adult	562 (98.1)
Treatment regimen (*n*, %)	
2HRZE + 4HR	486 (78.8)
6HRZE	73 (11.8)
2HRZE + 7HRE	53 (8.6)
6HRZE + Lfx	5 (0.8)
HIV infection (*n*, %)	
Positive	12 (1.9)
Negative	605 (98.1)
ART (*n*, %)	
Yes	12 (1.9)
NA	605 (98.1)

*Note:* H: isoniazid; R: rifampicin; Z: pyrazinamide; E: ethambutol; Lfx: levofloxacin; PCD: pulmonary clinically confirmed.

Abbreviations: EP = extra pulmonary, PBC = pulmonary bacteriologically confirmed.

**Table 4:** Prevalence of MDR-TB.

Total sample size (*n*) = 617	DR-TB (+)	Prevalence (95% CI)
	*N* = 16	2.6% (1.34%–3.86%)

**Table 5:** Association of DR-TB with sociodemographics and TB-related clinical parameters.

Variables	Drug resistance		*p* value
	Yes (%)	No (%)	
Age (continuous)	13.06 ± 20.39	38.21 ± 20.06	0.34
Age (category)			
Up to 40	8 (2.2)	361 (97.8)	0.418
More than 40	8 (3.2)	240 (96.8)	
Gender			
Male	6 (1.7)	384 (98.3)	0.103
Female	10 (3.8)	253 (96.2)	
Body mass index			
Less than 25	10 (2.2)	449 (97.8)	0.247
25 and more	6 (3.9)	148 (96.1)	
TB type			0.172
Pulmonary	9 (2.1)	419 (97.9)	
Extra pulmonary	7 (3.7)	182 (96.3)	
Under CB DOTS program			0.36
Yes	0	29 (100.0)	
No	16 (2.7)	572 (97.3)	
Treatment regimen			—
2HRZE + 4HR	15 (3.1)	471 (96.9)	
6HRZE	0	73 (100.0)	
2HRZE + 7HRE	1 (1.96)	52 (98.1)	
6HRZE + Lfx	0	5 (100.0)	
HIV infection			**0.002**
Positive	2 (16.7)	10 (83.3)	
Negative	14 (2.3)	591 (97.7)	
Current smoking status			**< 0.001**
Yes	7 (13.2)	46 (86.8)	
No	9 (1.6)	555 (98.4)	
Exposure to smoking			**< 0.001**
Yes	11 (8.2)	123 (91.8)	
No	478 (99)	5 (1.0)	
Current alcohol consumer			**0.006**
Yes	4 (8.9)	41 (91.1)	
No	12 (2.1)	560 (97.9)	

*Note:* H: isoniazid; R: rifampicin; Z: pyrazinamide; E: ethambutol; Lfx: livofloxacin. Statistical significance is indicated by *p* values less than 0.05, which are highlighted in bold.

**Table 6:** Bivariate and multivariate analyses for the determinants of DR-TB.

Variables	COR (95% CI)	*p* value	AOR (95% CI)	*p* value
Current smoking status				
Yes	9.384 (3.342–26.351)	**< 0.001** ^**∗**^	0.359 (0.094–1.375)	0.135
No	Ref			
Exposure to smoking				
Yes	8.550 (2.916–25.064)	**< 0.001** ^**∗**^	5.317 (1.394–20.274)	**0.014** ^**∗**^
No	Ref			
Current alcohol consumer				
Yes	4.553 (1.406–14.745)	**0.006** ^**∗**^	6.84 (2.071–22.58)	**0.002** ^**∗**^
No	Ref			
HIV infection				
Positive	8.443 (1.691–42.155)	**0.002** ^**∗**^	0.661 (0.082–5.329)	0.697
Negative	Ref			

^∗^Significant at < 0.05. The bold values only used for highlighting the significant value, which is less than 5%, i.e., *p* < 0.05.

## Data Availability

The data that support the findings of this study are available on request from the corresponding author. The data are not publicly available due to privacy or ethical restrictions.
